# Supplementary material for: RcsF-independent mechanisms of signaling within the Rcs Phosphorelay
Source: bioRxiv. 2024 Sep 3:2024.08.29.610257. Preprint. [Version 2] doi: 10.1101/2024.08.29.610257 (PMC11451591; doi:10.1101/2024.08.29.610257)
Supplement: Supplement 1 — Fig. S1: Signaling by putative RcsF-independent Rcs activators. A genomic library was transformed into an rcsF deletion strain (EAW34) containing the PrprA::mCherry reporter for Rcs signaling and the colonies were screened for increased fluorescence. The plasmids were isolated from colonies showing high fluorescence (indicating Rcs activation) and sequenced. The genes present in the candidate plasmids, representing two genomic regions, are shown here. A. The gene encoding the YedR/DrpB ORF (indicated here in red) was present in 13 out of 14 candidate plasmids, in 8 independent clones with different end points. B. The remaining candidate plasmid contained genes for spot42 sRNA (spf) and the YihA ORF. C. For this PrprA::mCherry assay, the strain AP 51 (rcsF::kan) overexpressing pBR-plac (V), pBR-plac-rseX or pBR-plac-spf was grown in MOPS minimal glycerol medium containing 100 μg/ml ampicillin and their fluorescence measured over time at 37°C. The RFU at OD 0.4 is plotted with the value for the vector in the absence of IPTG set to 1. The strains were induced with 100 μM IPTG. D. The AP51 strain containing pBAD33 or pBAD33-yihA (pAP3327) was grown in MOPS minimal glycerol medium with 25 μg/ml chloramphenicol and induced with 0.02% arabinose. Data from three independent experiments is plotted as mean with error bars indicating the standard deviation. Values were statistically analyzed using multiple unpaired t-tests. ‘ns’ indicates a P-value > 0.05 (non-significant). Fig. S2: Dependence of DsbA, DjlA, and DrpB on each other for Rcs activation: A. Effect of DjlA or DrpB deletion upon Rcs signaling in dsbA mutants: All the strains carry an rprA promoter fusion to mCherry (PrprA-mCherry) and the mCherry fluorescence acts as an indicator for Rcs activation. For the PrprA::mCherry assay, the cells were grown in MOPS minimal glucose medium at 37°C. The RFU at OD 0.4 for the WT uninduced was set to 1 and the relative induction compared to that depicted here (top panel). The ce [file media-1.pdf]

RcsF-independent mechanisms of signaling within the Rcs Phosphorelay

Anushya Petchiappan[1], Nadim Majdalani[1], Erin Wall[1, 2] and Susan Gottesman[1, *]

Supplementary Figures, S1-S9

Supplementary Tables:

S1: Strains

S2: Plasmids

S3: Primers

S4: gBlocks

References

Supplementary Figures:

Fig. S1:

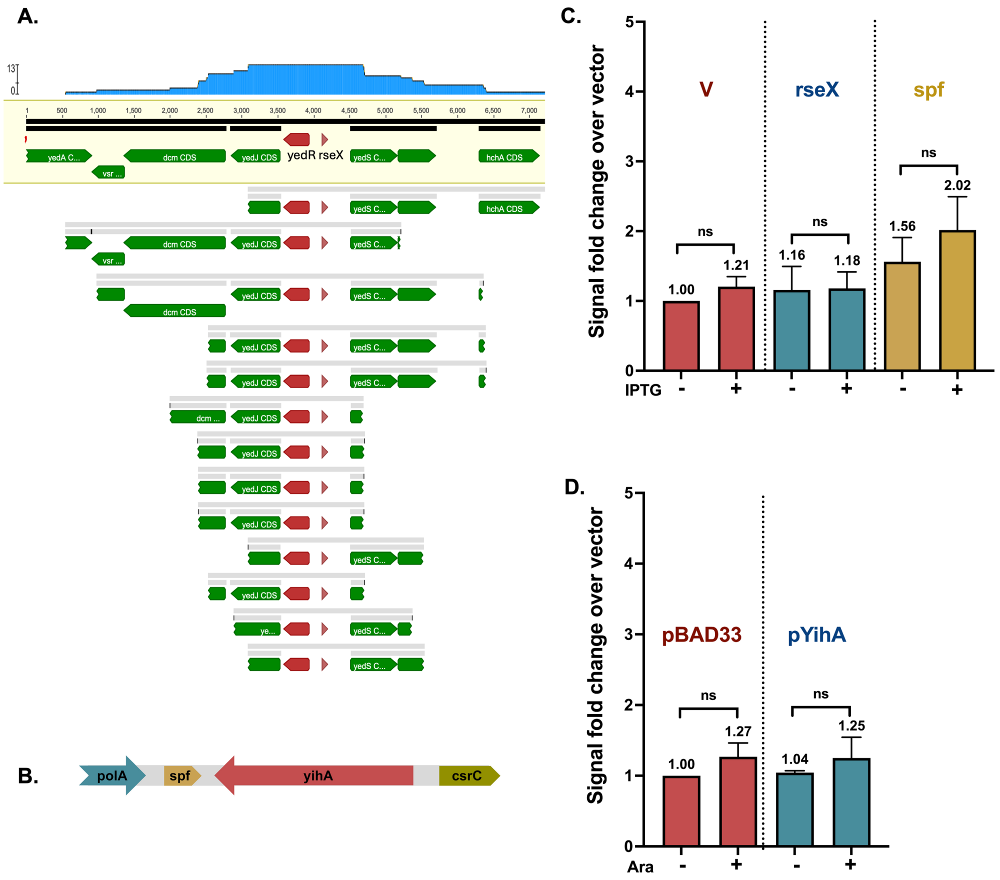

Fig. S2:

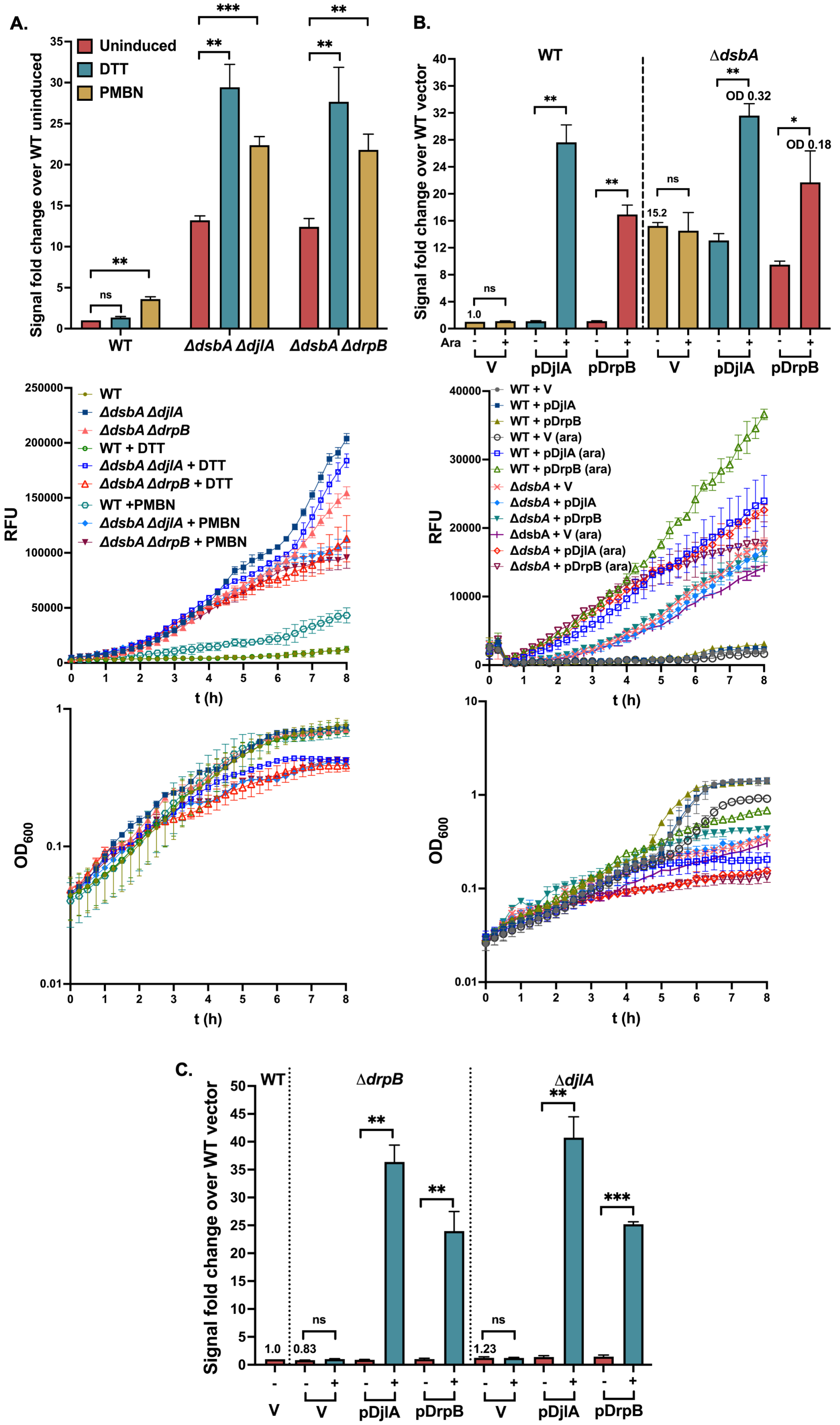
A.
Uninduced
DTT
PMBN
Signal fold change over WT uninduced
WT
ΔdsbA ΔdjlA
ΔdsbA ΔdrpB
ns
**
***
B.
WT
ΔdsbA
Signal fold change over WT vector
1.0
15.2
OD 0.32
OD 0.18
Ara
V
pDjlA
pDrpB
RFU
t (h)
WT + DTT
ΔdsbA ΔdjlA + DTT
ΔdsbA ΔdrpB + DTT
WT +PMBN
ΔdsbA ΔdjlA + PMBN
ΔdsbA ΔdrpB + PMBN
WT + V
WT + pDjlA
WT + pDrpB
WT + V (ara)
WT + pDjlA (ara)
WT + pDrpB (ara)
ΔdsbA + V
ΔdsbA + pDjlA
ΔdsbA + pDrpB
ΔdsbA + V (ara)
ΔdsbA + pDjlA (ara)
ΔdsbA + pDrpB (ara)
OD600
C.
ΔdrpB
ΔdjlA
0.83
1.23

Fig. S3:

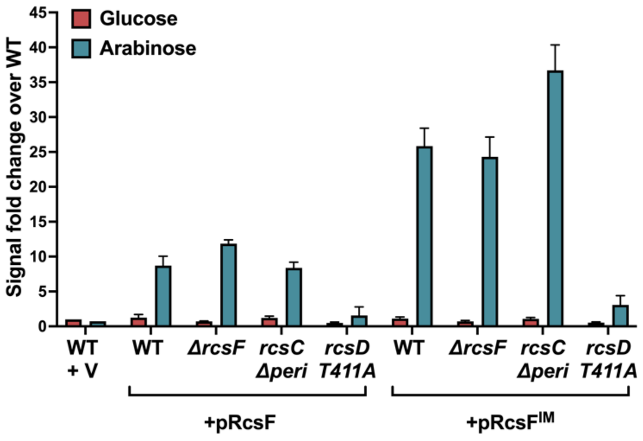
Glucose
Arabinose
Signal fold change over WT
45
40
35
30
25
20
15
10
5
0
WT + V
WT
ΔrcsF
rcsC Δperi
rcsD T411A
WT
ΔrcsF
rcsC Δperi
rcsD T411A
+pRcsF
+pRcsF^IM

Fig. S4:

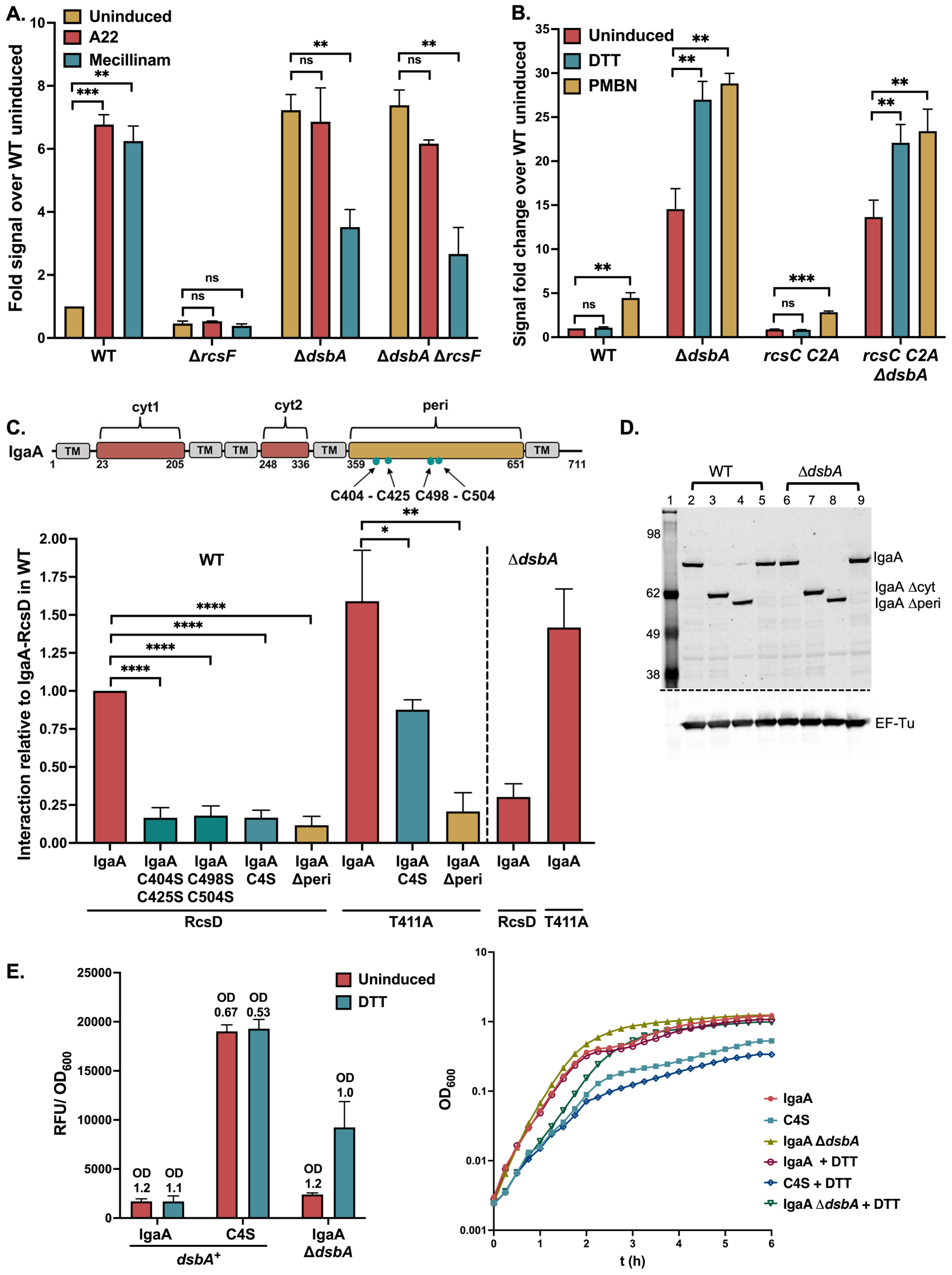
A.
Uninduced
A22
Mecillinam
Fold signal over WT uninduced
ns
WT
ΔrcsF
ΔdsbA
ΔdsbA ΔrcsF
B.
Uninduced
DTT
PMBN
Signal fold change over WT uninduced
WT
ΔdsbA
rcsC C2A
rcsC C2A ΔdsbA
C.
IgaA
cyt1
cyt2
peri
TM
C404 - C425 C498 - C504
Interaction relative to IgaA-RcsD in WT
WT
ΔdsbA
IgaA
IgaA C404S C425S
IgaA C498S C504S
IgaA C4S
IgaA Δperi
RcsD
T411A
D.
WT
ΔdsbA
IgaA
IgaA Δcyt
IgaA Δperi
EF-Tu
E.
Uninduced
DTT
RFU/ OD600
OD 1.2
OD 1.1
OD 0.67
OD 0.53
OD 1.2
OD 1.0
IgaA
C4S
IgaA ΔdsbA
dsbA+
OD600
t (h)
IgaA
C4S
IgaA ΔdsbA
IgaA + DTT
C4S + DTT
IgaA ΔdsbA + DTT

Fig. S5:

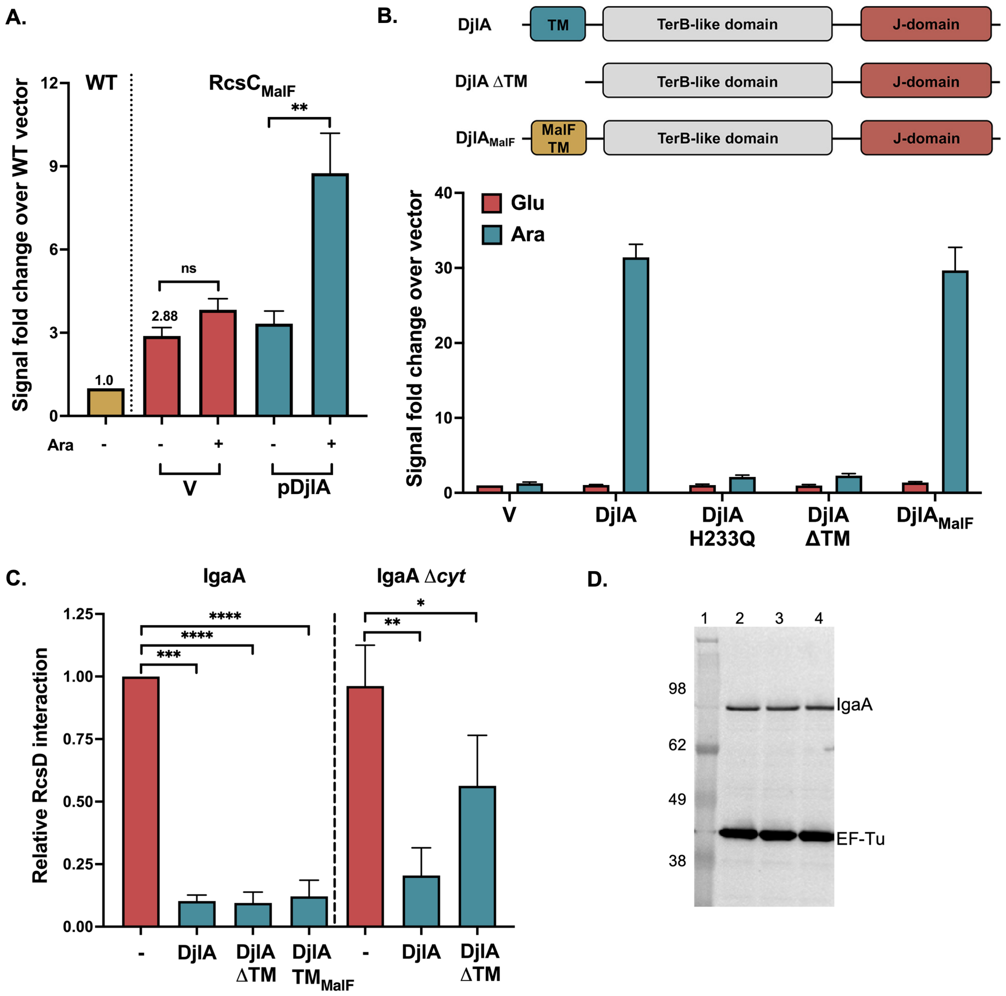
A.
WT
RcsC$_{MalF}$
Signal fold change over WT vector
1.0
2.88
ns
**
Ara
V
pDjlA
B.
DjlA
TM
TerB-like domain
J-domain
DjlA ΔTM
DjlA$_{MalF}$
MalF TM
Glu
Ara
Signal fold change over vector
V
DjlA
DjlA H233Q
DjlA ΔTM
DjlA$_{MalF}$
C.
IgaA
IgaA Δ*cyt*
Relative RcsD interaction
***
****
**
*
-
DjlA
DjlA ΔTM
DjlA TM$_{MalF}$
D.
1
2
3
4
98
62
49
38
IgaA
EF-Tu

Fig. S6:

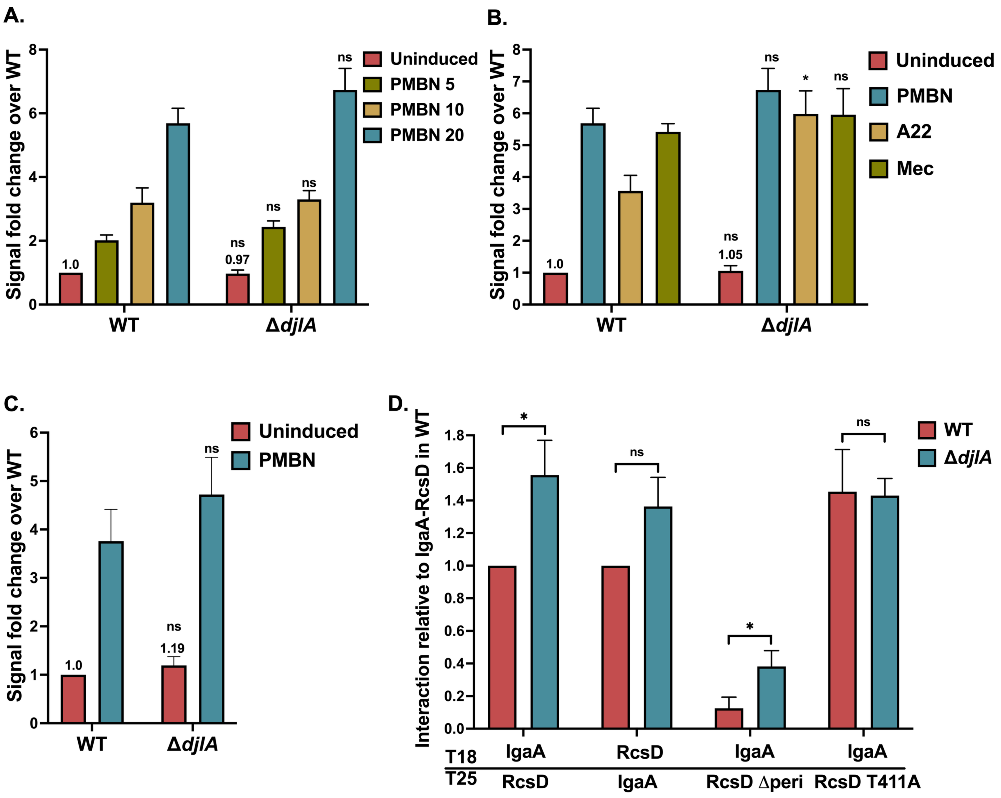
A.
Signal fold change over WT
Uninduced
PMBN 5
PMBN 10
PMBN 20
1.0
0.97
ns
WT
ΔdjlA
B.
Signal fold change over WT
Uninduced
PMBN
A22
Mec
1.0
1.05
ns
*
WT
ΔdjlA
C.
Signal fold change over WT
Uninduced
PMBN
1.0
1.19
ns
WT
ΔdjlA
D.
Interaction relative to IgaA-RcsD in WT
WT
ΔdjlA
*
ns
T18
T25
IgaA
RcsD
RcsD
IgaA
IgaA
RcsD Δperi
IgaA
RcsD T411A

Fig. S7:

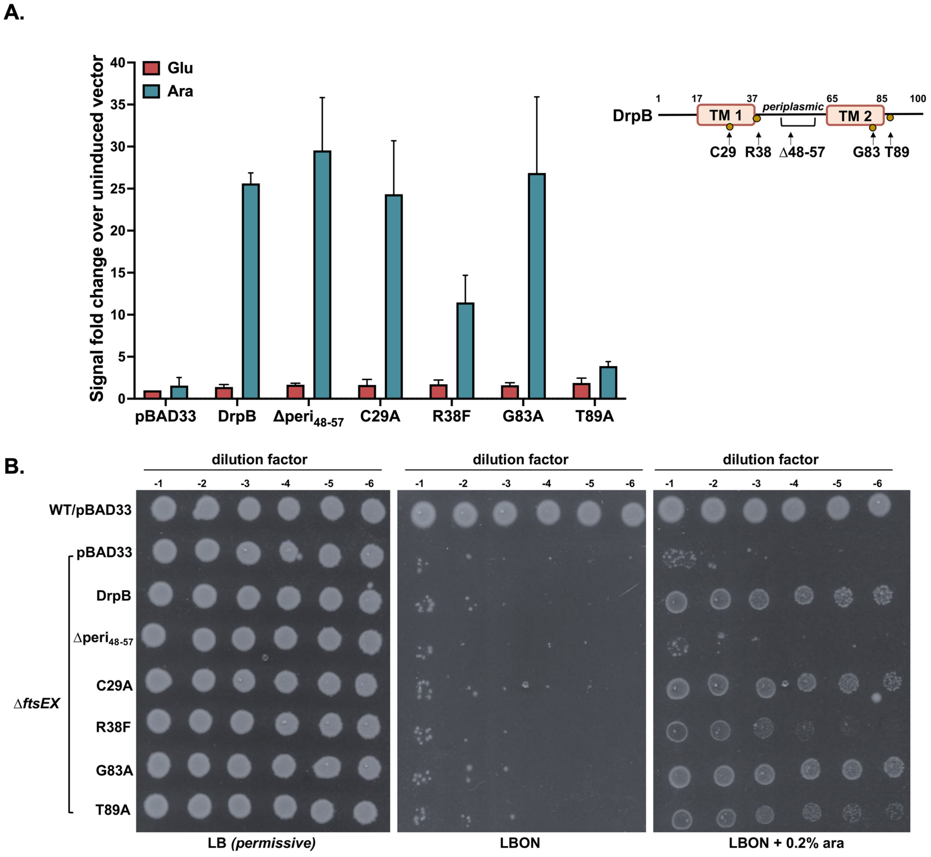
A.
Glu
Ara
Signal fold change over uninduced vector
0
5
10
15
20
25
30
35
40
pBAD33
DrpB
Δperi48-57
C29A
R38F
G83A
T89A
DrpB
1
17
37
periplasmic
65
85
100
TM 1
TM 2
C29
R38
Δ48-57
G83
T89
B.
dilution factor
-1
-2
-3
-4
-5
-6
WT/pBAD33
pBAD33
DrpB
Δperi48-57
C29A
R38F
G83A
T89A
ΔftsEX
LB (permissive)
LBON
LBON + 0.2% ara

Fig. S8:

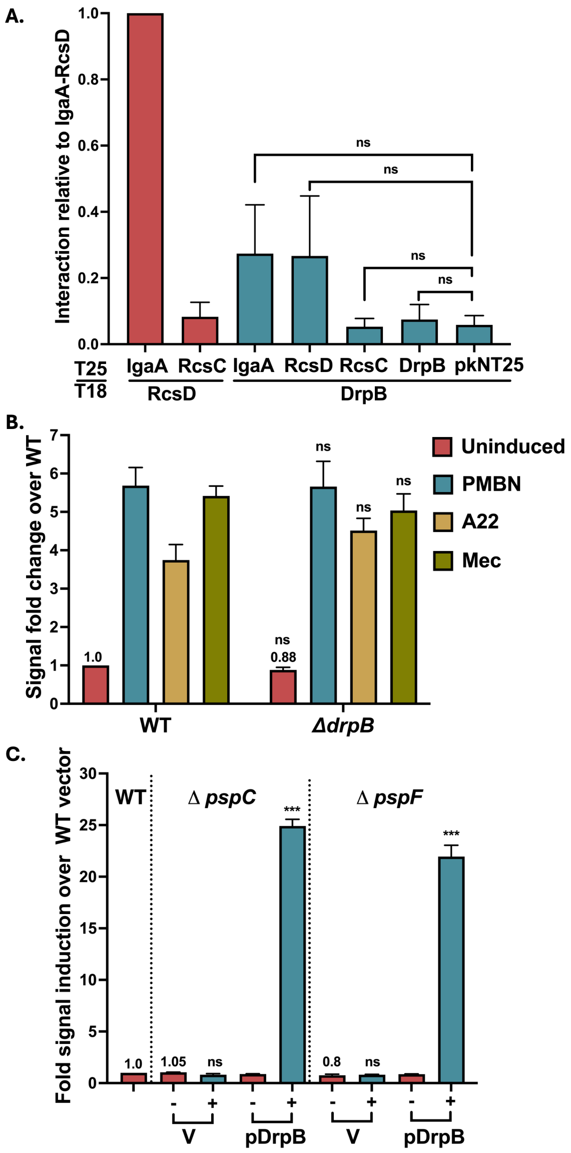

A.
Interaction relative to IgaA-RcsD
1.0
0.8
0.6
0.4
0.2
0.0
ns
ns
ns
ns
T25
T18
IgaA
RcsC
IgaA
RcsD
RcsC
DrpB
pkNT25
RcsD
DrpB
B.
Signal fold change over WT
7
6
5
4
3
2
1
0
Uninduced
PMBN
A22
Mec
1.0
ns
0.88
ns
ns
ns
ns
WT
ΔdrpB
C.
Fold signal induction over WT vector
30
25
20
15
10
5
0
WT
Δ pspC
Δ pspF
***
***
1.0
1.05
ns
0.8
ns
-
+
-
+
-
+
-
+
V
pDrpB
V
pDrpB

Fig. S9:

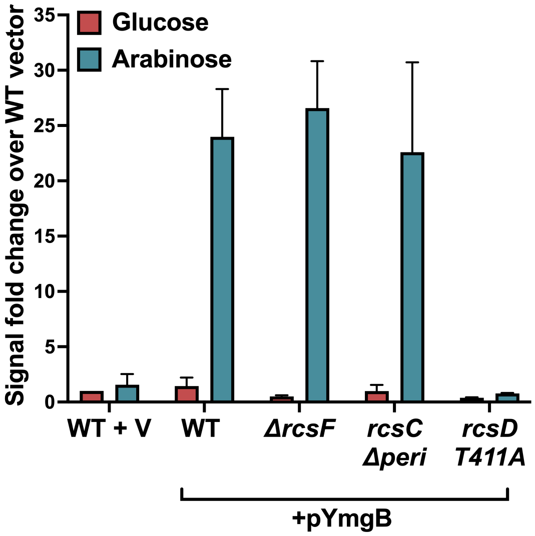
Glucose
Arabinose
Signal fold change over WT vector
35
30
25
20
15
10
5
0
WT + V
WT
ΔrcsF
rcsC Δperi
rcsD T411A
+pYmgB

<u>**Supplementary Tables**</u>

Table S1: List of strains used in this study

Strains were constructed by recombineering or P1 transduction with selectable markers (Table S1). Recombineering was done in strains carrying a chromosomal mini-λ Red system (*miniλ::tet*) or a plasmid-borne Red system (pSIM27). Some strains were generated by direct P1 transduction from the corresponding mutant strains in the Keio collection [1].

Name	Genotype	Method of construction or reference
MG1655	Wild-type *E. coli* K-12	Lab collection
BTH101	*F-, cya-99, araD139, galE15, galK16, rpsL1 (Str^r), hsdR2, mcrA1, mcrB1*	[2]
NEB DH5-alpha F'IQ	F´ *proA$^+$B$^+$ lacI^q Δ(lacZ)M15 zzf::Tn10* (Tet^R) / *fhuA2Δ(argF-lacZ)U169 phoA glnV44 Φ80Δ(lacZ)M15 gyrA96 recA1 relA1 endA1 thi-1 hsdR17*	New England Biolabs
SG20382	*rcsB11*::Tn10 (*tet^r*)	[3]
DH300	$P_{rprA142}$-*lacZ*	[4]
DH311	$P_{rprA142}$-*lacZ, rcsB311::kan*	[4]
DH339	$P_{rprA142}$-*lacZ, yojN::kan* (*rcsD542*)	[5]
DH375	$P_{rprA142}$-*lacZ, rcsC C111A C154A with atoS::kan*	DH300 + P1 (NM344a)$^{\#}$
DJ480	MG1655 *lacX74*	[6]
TKC	*tetA, cat, kan*	[7]
NC397	*W3110 pglΔ8 gal$_{490}$ λ cI857(cro-bioA)$_{Δ}$ lacI^{o} <>kan-Ter<>cat sacB <>lacZYA*	[8]
HK307	MC1000 *dsbA::kan*	Beckwith lab
EC251	MG1655	[9]
EC855	MG1655 *ftsE::kan*	Weiss lab
EC1215	*ΔftsEX<>frt*	[9]
NM7	*ΔrcsF12::cat-sacB*	[10]
NM300	DJ480 *mini-λ-tet*	[11]
NM338	DJ480 *rcsC C111A- cat-sacB*	NM300 + linear transformation

NM340	DJ480 *rcsC C111A*	NM338 + single-stranded Cys111Ala replacement primer
NM344a	DJ480 *rcsC C111A C154A with atoS*::*kan*	NM355 + linear transformation
NM350	DJ480 *rcsC C111A with atoS*::*kan*	NM340 + linear transformation
NM355	DJ480 *ΔrcsC154-atoS::cat*	NM300 linear transformation
NM358	DJ480 *mini-λ-tet, rcsB311::kan*	NM300 + P1 (DH311)
NM364	DJ480 *mini-λ-tet, rcsB311::kan, Δwza-ΔcpsB::zeo*	NM358 electroporated with NM1201 PCR product (cpsB-zeo.R and wza-zeo.F)
NM1201	MG1655 *ybeW::zeo*	[12]
EAW1	*rcsB11*::Tn10, *cya*	[10]
EAW2	*rcsC32*::Tn10, *cya*	[10]
EAW4	*ΔrcsF12*::*cat-sacB*, *cya*	[10]
EAW8	*ΔaraBAD*::$P_{rprA142}$-mCherry, *ΔaraE*p P_{CP6}::*gent*::P_{cp18}-*araE*	[10]
EAW18	*ΔaraBAD*::$P_{rprA142}$-mCherry, *rcsC*::Tn10, *ΔaraE*p P_{cp6}*gent*::P_{cp18}-*araE*	[10]
EAW19	*ΔaraBAD*::$P_{rprA142}$-mCherry, *rcsD*541(::FRT), *ΔaraEp* P_{cp6}*gent*::P_{cp18}-*araE*	[10]
EAW25	*ΔaraBAD*::$P_{rprA142}$-mCherry, *ΔaraE*p P_{CP6}::*gent*::P_{cp18}-*araE, Δwza-ΔcpsB::zeo*	EAW8 + P1 from NM364 (screened for *Δwza-ΔcpsB::zeo*)
EAW31	*ΔaraBAD*::$P_{rprA142}$-mCherry, *ΔaraE*p P_{cp6}*gent*::P_{cp18}-*araE, rcsB*::*kan*	[10]
EAW32	*ΔaraBAD*::$P_{rprA142}$-mCherry, *ΔaraE*p P_{cp6}*gent*::P_{cp18}-*araE*, D*rcsF12*::*cat-sacB*	[10]
EAW34	*ΔaraBAD*::$P_{rprA142}$-mCherry, *ΔaraE*p P_{CP6}::*gent*::P_{cp18}-*araE, Δwza-ΔcpsB::zeo*, *ΔrcsF12::cat-sacB*	EAW25 + P1 (NM7)
EAW70	*ΔaraBAD*::$P_{rprA142}$-mCherry, *ΔaraE*p P_{cp6}*gent*::P_{cp18}-*araE*, *ΔrcsC51::rcsCΔ48-314*	[10]
EAW72	*ΔaraBAD*::$P_{rprA142}$-mCherry, *ΔaraE*p P_{cp6}*gent*::P_{cp18}-*araE*, *ΔrcsC51::rcsC*$_{1-19}$-*malF*$_{2-59}$-*rcsC*$_{334-C}$	[10]
EAW120	*ΔaraBAD*::$P_{rprA142}$-mCherry, *ΔaraE*p P_{cp6}*gent*::P_{cp18}-*araE*, *rcsD841**	[10]

EAW121	*ΔaraBAD*::P*rprA142*-mCherry, *ΔaraE*p P*cp6gent*::P*cp18*-*araE*, *rcsDT411A*	[10]
EAW62	*ΔaraBAD*::P*rprA142*-mCherry, *ΔaraE*p P*CP6*::*gent*::P*cp18*-*araE*, *dsbA::kan*	EAW8 + P1 (HK307)
EAW63	*ΔaraBAD*::P*rprA142*-mCherry, *rcsC*::Tn10, *ΔaraE*p P*cp6gent*::P*cp18*-*araE*, *dsbA::kan*	EAW18 + P1 (HK307)
EAW67	*ΔaraBAD*::P*rprA142*-mCherry, *ΔaraE*p P*CP6*::*gent*::P*cp18*-*araE*, *dsbA::kan*, Δ*rcsF12::cat-sacB*	EAW62 + P1 (NM7)
EAW74	*ΔaraBAD*::P*rprA142*-mCherry, *ΔaraE*p P*cp6gent*::P*cp18*-*araE*, *ΔrcsC51::rcsCΔ48-314*, *dsbA::kan*	EAW70 + P1 (HK307)
EAW90	*ΔaraBAD*::P*rprA142*-mCherry, *ΔaraE*p P*cp6gent*::P*cp18*-*araE*, *rcsD541(::FRT)*, *ΔigaA*::*kan-araC-kid*	[10]
AP11	*ΔaraBAD*::P*rprA142*-mCherry, *ΔaraE*p P*CP6*::*gent*::P*cp18*-*araE*, Δ*dsbA*	EAW62 + pcp20
AP12	*ΔaraBAD*::P*rprA142*-mCherry, *ΔaraE*p P*CP6*::*gent*::P*cp18*-*araE*, Δ*dsbA*, *rcsB311::kan*	AP11 + P1 (DH311)
AP13	*ΔaraBAD*::P*rprA142*-mCherry, *ΔaraE*p P*CP6*::*gent*::P*cp18*-*araE*, Δ*dsbA*, *yojN::kan (rcsD542)*	AP11 + P1 (DH339)
AP14	*ΔaraBAD*::P*rprA142*-mCherry, *ΔaraE*p P*cp6gent*::P*cp18*-*araE*, *rcsDT411A*, *dsbA::kan*	EAW121 + P1 (HK307)
AP41	*ΔaraBAD*::P*rprA142*-mCherry, *ΔaraE*p P*CP6*::*gent*::P*cp18*-*araE*, *drpB::kan*	EAW8 + P1 (Keio JW1946)
AP46	*ΔaraBAD*::P*rprA142*-mCherry, *ΔaraE*p P*CP6*::*gent*::P*cp18*-*araE*, *djlA::kan*	EAW8 + P1 (Keio JW0054)
AP50	*ΔaraBAD*::P*rprA142*-mCherry, *ΔaraE*p P*CP6*::*gent*::P*cp18*-*araE*, *rcsB11::Tn10 (tetr)*	EAW8 + P1 (SG20382)
AP51	D*araBAD*::P*rprA142*-mCherry, D*araE*p P*CP6*::*gent*::P*cp18*-*araE*, *rcsF::kan*	EAW8 + P1 (Keio JW0192)
AP57	*dsbA::kan, cya*	BTH101 + P1 (HK307)
AP58	*ΔdsbA, cya*	AP57 + pcp20
AP71	*ΔaraBAD*::P*rprA142*-mCherry, *ΔaraE*p P*CP6*::*gent*::P*cp18*-*araE*, Δ*dsbA*, *drpB::kan*	AP11 + P1 (Keio JW1946)
AP72	*ΔaraBAD*::P*rprA142*-mCherry, *ΔaraE*p P*CP6*::*gent*::P*cp18*-*araE*, Δ*dsbA*, *djlA::kan*	AP11 + P1 (Keio JW0054)
AP113	*ΔaraBAD*::P*rprA142*-mCherry, *ΔaraE*p P*CP6*::*gent*::P*cp18*-*araE*, *pspC::kan*	EAW8 + P1 (Keio JW1299)

AP114	*ΔaraBAD*::$P_{rprA142}$-mCherry, *ΔaraE*p P_{CP6}::*gent*::P_{cp18}-*araE, pspF::kan*	EAW8 + P1 (Keio JW1296)
AP154	*ΔaraBAD*::$P_{rprA142}$-mCherry, *ΔaraE*p P_{CP6}::*gent*::P_{cp18}-*araE, ftsE::kan*	EAW8 + P1 (EC855)
AP155	*ΔaraBAD*::$P_{rprA142}$-mCherry, *ΔaraE*p P_{CP6}::*gent*::P_{cp18}-*araE, rcsB11::Tn10* (tet^r), *ftsE::kan*	AP50 + P1 (EC855)
AP158	*rcsB311*::*kan*	EC251 + P1 (DH311)
AP159	*ΔftsEX<>frt, rcsB311*::*kan*	EC1215 + P1 (DH311)
AP168	*ΔaraBAD*::$P_{rprA142}$-mCherry, *ΔaraE*p P_{cp6}*gent*::P_{cp18}-*araE*, *rcsD541(::FRT)*, *ΔigaA::igaA C404S C424S C498S C504S*	EAW90 recombination with PCR product of pEAW1C4S template using oligos EAW213 and EAW214
AP169	*ΔaraBAD*::$P_{rprA142}$-mCherry, *rcsD541(::FRT)*, *ΔaraEp* P_{cp6}*gent::*P_{cp18}-*araE*, *dsbA::kan*	EAW19 + P1 (EAW62)
AP172	*ΔaraBAD*::$P_{rprA142}$-mCherry, *ΔaraE*p P_{CP6}::*gent*::P_{cp18}-*araE, rcsC C111A C154A with atoS::kan*	EAW8 + P1 (DH375)
AP173	*ΔaraBAD*::$P_{rprA142}$-mCherry, *ΔaraE*p P_{CP6}::*gent*::P_{cp18}-*araE, rcsC C111A C154A with atoS::kan, ΔdsbA*	AP11 + P1 (DH375)

[#]**Construction of NM344a:**

NM344a was constructed in two steps. In the first step, the region between *atoS* and *rcsCcys154* in NM300 was replaced by recombineering a *cat* resistance cassette using the primers *ΔatoS-rcsC154.CmF* and *ΔatoS-rcsC154.CmR* and the TKC strain used as a template. This generated NM355. In a second parallel step, NM338 was generated by recombineering the PCR product (primers Cys111-ala_cat and Cys111-ala_sacB) from NC397 into NM300. Next, NM340 was generated by linear transformation of the single-stranded oligo Cys111Ala replacement primer into NM338. In this strain NM340, a *kan* resistance cassette was then inserted between *rcsCcys111* and *atoS* using primers RcsC-KAN-AtoS.F and RcsC-KAN-AtoS.R. This generated strain NM350. A 2-kb fragment from the *kan* cassette to the rcsC154 nucleotide was amplified from NM350 using the primers atoS_RcsCys154 (containing the Cys154Ala mutation) and RcsC-KAN-AtoS.R was used to transform NM355. This generated strain NM344a.

Table S2: List of plasmids used in this study

Plasmids were constructed by the Gibson assembly method using the In-fusion HD Cloning kit (Takara Bio USA) [13]. Site-directed mutagenesis (SDM) in the genes was carried out using the QuikChange Site-directed mutagenesis kit (Agilent).

Name	Description	Method of construction/ Reference
pBAD24	Vector for protein expression regulated by the arabinose operon (Amp^r)	[14]
pBAD33	Vector for protein expression regulated by the arabinose operon (Chl^r)	[14]
pCP20	Plasmid with temperature-sensitive origin of replication, encoding the FLP recombinase	[15]
pUT18	Vector encoding the Cya T18 fragment under lac promoter control (Amp^r)	[16]
pKNT25	Vector encoding the Cya T18 fragment under lac promoter control (Kan^r)	[17]
pSIM27	Plasmid with temperature-sensitive origin of replication, encoding l-Red cI857, gam-beta-exo	https://ncifrederick.cancer.gov/recombineering/strains-plasmids-and-primers
pEAW1	IgaA with T18 tag at C-terminal cloned in pUT18	[10]
pEAW1cyt1	IgaA *Δ*36-181 with T18 tag at C-terminal cloned in pUT18	[10]
pEAW1peri	IgaA *Δ*384-649 with T18 tag at C-terminal cloned in pUT18	[10]
pEAW2	IgaA with T25 tag at C-terminal cloned in pKNT25	[10]
pEAW6	RcsC with T25 tag at C-terminal cloned in pKNT25	[10]
pEAW7	RcsD with T18 tag at C-terminal cloned in pUT18	[10]
pEAW8	RcsD with T25 tag at C-terminal cloned in pKNT25	[10]
pEAW8peri	RcsD *Δ*45-304 with T25 tag at C-terminal cloned in pKNT25	[10]
pEAW8T	RcsD T411A with T25 tag at C-terminal cloned in pKNT25	[10]
pEAW11	RcsD cloned in pBAD24	[10]
pEAW11T	RcsD T411A cloned in pBAD24	[10]

pPSG961	DjlA cloned in pBAD33	[18] (Gift from A. Jacq?)
pDSW1977	DrpB cloned in pBAD33	[9] (Gift from David Weiss)
pBR-plac	pBR322 derivative plasmid for sRNA overexpression under an artificial P_{lac} promoter	[19]
p-rseX	rseX sRNA cloned in pBR-plac	[20]
p-spf	spot42 sRNA cloned in pBR-plac	[20]
pNM654	IgaA cloned in pBAD24	IgaA amplified from genomic DNA (yrfF_pBAD24F and R); pBAD24 digested with *EcoRI* and *HindIII*
pNM656	IgaA C425S cloned in pBAD24	pNM654 template with primers yrfF Cys425Ser.F and Cys425Ser.R (SDM)
pNM665	IgaA C425S C498S C504S cloned in pBAD24	pNM656 template with primers yrfF Cys498-504Ser.F and Cys498-504Ser.R (SDM)
pNM671	IgaA C404S C425S C498S C504S cloned in pBAD24	pNM665 template with primers yrfF Cys404Ser.F and Cys404Ser.R (SDM)
pAP101	IgaA *Δ*36-181 Δ263-329 with T18 tag at C-terminal cloned in pUT18	pEAW1cyt1 template with primers EW209 and EW210
pAP102	IgaA C404S C425S with T18 tag at C-terminal cloned in pUT18	pAP104 template with primers AP691 and AP692 (SDM)
pAP103	IgaA C498S C504S with T18 tag at C-terminal cloned in pUT18	pAP105 template with primers AP587 and AP588 (SDM)
pAP104	IgaA C404S with T18 tag at C-terminal cloned in pUT18	pEAW1 template with primers AP351 and AP352 (SDM)
pAP105	IgaA C498S with T18 tag at C-terminal cloned in pUT18	pEAW1 template with primers AP693 and AP694 (SDM)
pEAW1C4S	IgaA C404S C425S C498S C504S (C4S) with T18 tag at C-terminal cloned in pUT18	Insert from pNM671 (EW6 and EW7); pUT18 linearized with EW1 and EW2
pAP1401	IgaA with T18 tag at C-terminal and DjlA cloned downstream under the same promoter in pUT18	Insert from gBlock AP_GJ1; pEAW1 linearized with AP375 and AP376
pAP1402	IgaA with T18 tag at C-terminal and DjlA H233Q cloned downstream under the same promoter in pUT18	pAP1401 template with primers AP459 and AP460 (SDM)
pAP1403	IgaA with T18 tag at C-terminal and DjlA Δ1-31 (ΔTM) cloned downstream under the same promoter in pUT18	pAP1401 template with primers AP495 and AP496 (SDM)

pAP1404	IgaA with T18 tag at C-terminal and DjlA TM_{MalF} cloned downstream under the same promoter in pUT18	Insert from gBlock AP_GJMF; pEAW1 linearized with AP375 and AP376
pAP407	DrpB with T18 tag at C-terminal cloned in pUT18	Insert from pDSW1977 (AP241 and AP242); pUT18 linearized with EW1 and EW2
pAP408	DrpB with T25 tag at C-terminal cloned in pKNT25	Insert from pDSW1977 (AP241 and AP242); pUT18 linearized with EW1 and EW2
pAP804	RcsD T411A Δ45-304 with T25 tag at C-terminal cloned in pKNT25	pEAW8peri template with primers T411A F and T411A R (SDM)
pAP3301	DrpB Δ48-59 cloned in pBAD33	pDSW1977 template with primers AP321 and AP322 (SDM)
pAP3304	DrpB C29A cloned in pBAD33	pDSW1977 template with primers AP323 and AP324 (SDM)
pAP3305	DrpB R38F cloned in pBAD33	pDSW1977 template with primers AP325 and AP326 (SDM)
pAP3306	DrpB G83A cloned in pBAD33	pDSW1977 template with primers AP327 and AP328 (SDM)
pAP3307	DrpB T89A cloned in pBAD33	pDSW1977 template with primers AP329 and AP330 (SDM)
pAP3311	DjlA Δ1-31 (ΔTM) cloned in pBAD33	pPSG961 template with primers AP563 and AP564
pAP3312	DjlA TM_{MalF} cloned in pBAD33	Insert from gBlock AP_GJMF (AP565 and AP566); pBAD33 linearized with AP559 and AP560
pAP3315	DjlA H233Q cloned in pBAD33	pPSG961 template with primers AP459 and AP460 (SDM)
pAP3325	YmgB/AriR cloned in pBAD33	Insert from gBlock AP_GymgB; pBAD33 linearized with AP559 and AP560
pAP3327	YihA cloned in pBAD33	Insert from gBlock AP_GyihA; pBAD33 linearized with AP559 and AP560
pAP3340	RcsF cloned in pBAD33	Insert from gBlock AP_GF33; pBAD33 linearized with AP559 and AP560
pAP3341	RcsF S17D M18Q cloned in pBAD33	pAP3340 template with primers AP501 and AP502 (SDM)

Table S3: List of primers used in this study

Name	Sequence (5'-3')
EW1	AAT CAT GGT CAT AGC TGT TTC CTG TGT GAA ATT G
EW2	AGC TTG CAT GCC TGC AGG TCG AC
EW6	GCT ATG ACC ATG ATT AGC ACC ATT GTG ATT TTT TTA GCT GCT TTG CTG
EW7	GCA GGC ATG CAA GCT TTC GAT AAG GCT TTC TGA AGG GGT GAT C
EW209	TAA CGG AAA GTT TTT ACC GCG CAG ACA ATG AAT TTC CCG C
EW210	AAA AAC TTT CCG TTA CAG CAC TGG CTG CGC
EW213	ACC ACG CCT GAC AGA CTA AGT AAG ATG GGG AAA GCA TGA GCA CCA TTG TGA TTT TTT TAG CTG CTT TGC
EW214	GAC AGG GTA GCA TAA CCT GCC GCG CAA ACG TGT TAT TCG ATA AGG CTT TCT GAA GGG GTG ATC AGT TG
ΔatoS-rcsC154.CmF	GCGTTCCACTGGCATATCACGCAGACCGAAATTGGCCATAAAATGAGACGTTG ATCGGCACG
ΔatoS-rcsC154.CmR	GGTTAAGGTGATGATTTCTCGGCGGTGTATCATATTCCAGACCAGCAATAGACAT AAGCGGGC
Cys111-ala_cat	CAGGCGGATGTGCCTGCGTTTGAACCGCTGTTTGCCGACTCCGATGCAAAATG AGCAGTTAGTCGGCAC
Cys111-ala_sacB	CGCCAATGACTCCAGAGAACCTCGCCAGGTGTTACTCATGTAGACTGCAAAGG GAAAACTGTCCATATG
Cys111Ala replacement primer	CAGGCGGATGTGCCTGCGTTTGAACCGCTGTTTGCCGACTCCGATGCTTCCGC AATGAGTAACACCTGGCGAGGTTCTCTGGAGTCATTGGCG
RcsC-KAN-AtoS.F	GGTAGCGGTAAAAGCGTGTTACCGCAATGTTCTCTCTTCTGTGTAGGCTGGAGC TGCTTCG
RcsC-KAN-AtoS.R	GGTTAAGGTGATGATTTCTCGGCGGTGTATCATATTCCAGATTCCGGGGATCCG TCGACC
atoS_RcsCys154	CGCGTTCCACTGGCATATCACGCAGACCGAAATTGGCCATAGCGAGGTTATCG CTGCCGATTAAAAATAC
cpsB-zeo.R	ATT TAC ACC GCG GTT TCG CAT TCA TTG CCT GAT GCG ACG TAA AAA AAG CCC GCT CAT TAG
wza-zeo.F	GTG CAC AGG ATA ATT ACT CTG CCA AAG TGA TAA ATA AAC AGT TGA CAA TTA ATC ATC GGC
yrfF_pBAD24F	CAG GAG GAA TTC ATG AGC ACC ATT GTG ATT TTT TTA GC

yrfF_pBAD24R	ACA GCC AAG CTT TTA TTC GAT AAG GCT TTC TG
yrfF Cys425Ser.F	TTT TTA CCG TTT GAC AGC TCG CAG ATC ATC T
yrfF Cys425Ser.R	AGA TGA TCT GCG AGC TGT CAA ACG GTA AAA A
yrfF Cys498-504Ser.F	AAG ACA GCG GAT TTA AGT TCT GCC AAA GAT GAC TGA GTG CGA CTG AAA AAT
yrfF Cys498-504Ser.R	ATT TTT CAG TCG CAC TCA GTC ATC TTT GGC AGA ACT TAA ATC CGC TGT CTT
yrfF Cys404Ser.F	AGC GGT ACG GGA ATG AGT AAT ATT CGA ACT T
yrfF Cys404Ser.R	AAG TTC GAA TAT TAC TCA TTC CCG TAC CGC T
AP241	GCT ATG ACC ATG ATT GAA TAC GGT TCG ACA AAG ATG GAA GAG AGA CTC T
AP242	GCA GGC ATG CAA GCT TTC ATA GCG TCT GCT ACG TGC GG
AP321	GGG AAT CCG GCT CAT CAC CCA GAT GTA AC
AP322	ATG AGC CGG ATT CCC TCT ACC GCG GGA AAG TGG TTA GGG
AP323	CAT TGC CCA GAC GAA ATA GCC CGC CCA TGT ATA GAA AGC CCA CAA
AP324	TTG TGG GCT TTC TAT ACA TGG GCG GGC TAT TTC GTC TGG GCA ATG
AP325	CTC ATC ACC CAG ATG TAA AAC GCC ATT GCC CAG ACG AA
AP326	TTC GTC TGG GCA ATG GCG TTT TAC ATC TGG GTG ATG AG
AP327	ACC AGG CAA TGC TGG CTA ACA ATG CCC CG
AP328	CGG GGC ATT GTT AGC CAG CAT TGC CTG GT
AP329	GGA CGA GGT CGG GCG TAC CAG GCA ATG
AP330	CAT TGC CTG GTA CGC CCG ACC TCG TCC
AP351	CGG AAG TTC GAA TAT TAG ACA TTC CCG TAC CGC TA
AP352	TAG CGG TAC GGG AAT GTC TAA TAT TCG AAC TTC CG
AP375	TTA TAT CGA TTG GCG TTC CAC TGC G
AP376	CTA AGT AAT ATG GTG CAC TCT CAG TAC AAT CTG CTC
AP459	CAGCTTATCGGGCTGGTGTTCACTCATCAGCTTACG
AP460	CGTAAGCTGATGAGTGAACACCAGCCCGATAAGCTG

AP495	CCC TGG ACC AAC GCC TTT CCG TTA CAG CAC TGG CTG CGC AGT AC
AP496	ATG TTT GAT AAA GCC CGT AGC CGT AAA ATG G
AP501	CGA CAG GGG ATC TGC TTA ACT GGT CAC AGC CGC TTA GCA TGA GTG
AP502	CAC TCA TGC TAA GCG GCT GTG ACC AGT TAA GCA GAT CCC CTG TCG
AP559	GAG CTC GAA TTC GCT AGC CCA AAA AAA CG
AP560	AAG CTT GGC TGT TTT GGC GGA TGA G
AP563	GGC TTT ATC AAA CAT ATA TTC CCC AGA TCG ACA CAC GGA TG
AP564	ATG TTT GAT AAA GCC CGT AGC CGT AAA ATG G
AP565	AGC GAA TTC GAG CTC AGC AGG AGG AAT TCA ATG GAT GTC ATT AAA AAG AAA C
AP566	AAA ACA GCC AAG CTT TCA TTT AAA CCC TTT CTG CTG CTT TAT CAG
AP587	CAT TTT TCA GTC GCA CAC TGT CAT CTT TGG CAG AAC A
AP588	TGT TCT GCC AAA GAT GAC AGT GTG CGA CTG AAA AAT G
AP691	CCA GAT GAT CTG CGA GCT GTC AAA CGG TAA AAA AG
AP692	CTT TTT TAC CGT TTG ACA GCT CGC AGA TCA TCT GG
AP693	CAT CTT TGG CAG AAG ATA AAT CCG CTG TCT TCA GTA C
AP694	GTA CTG AAG ACA GCG GAT TTA TCT TCT GCC AAA GAT G

TableS4: List of gBlocks used in this study

gBlock name	Sequence
**AP_GymgB**	AGCGAATTCGAGCTCAGCAGGAGGAATTCAATGCTTGAAGATACTACAATTCAT<br>AATGCAATAACTGATAAAGCGTTAGCAAGTTACTTTCGCAGTTCGGGTAATTTGT<br>TAGAAGAAGAATCAGCAGTGTTAGGGCAGGCTGTCACCAATTTAATGCTTTCAG<br>GCGATAATGTTAATAATAAAAATATTATCTTAAGTCTGATACACTCCTTGGAAAC<br>AACAAGTGATATTCTCAAAGCTGATGTGATTAGAAAAACACTGGAAATCGTGTT<br>GCGATACACAGCTGATGATATGTAAGCTTGGCTGTTTT
**AP_GyihA**	AGCGAATTCGAGCTCAGCAGGAGGAATTCAATGACTAATTTGAATTATCAACAGACGCA<br>TTTTGTGATGAGTGCGCCTGATATTCGCCACCTACCTTCCGATACCGGAATTGAAGTG<br>GCTTTTGCAGGCCGTTCCAACGCAGGTAAATCCAGCGCGCTGAACACGCTGACTAA<br>CCAGAAAAGCCTGGCTCGTACCTCAAAAACCCCAGGGCGCACCCAGCTTATCAACC<br>TGTTTGAAGTGGCTGACGGCAAGCGTCTGGTTGACTTGCCTGGGTACGGTTATGCGG<br>AAGTCCCGGAAGAGATGAAGCGCAAATGGCAGCGTGCGCTCGGCGAATACCTCGAA<br>AAACGTCAGAGCCTGCAAGGTCTGGTGGTGCTAATGGATATTCGCCATCCGCTGAAA<br>GATTTGGATCAGCAGATGATTGAGTGGGCGGTAGACAGCAATATCGCCGTTCTGGTG<br>CTGCTGACCAAAGCGGACAAACTGGCAAGCGGCGCACGTAAAGCGCAATTGAATAT<br>GGTGCGTGAAGCTGTACTGGCGTTTAACGGTGATGTGCAGGTTGAAACGTTTTCTTCG<br>TTGAAGAAACAAGGCGTGGACAAGCTGCGGCAGAAACTGGATACCTGGTTTAGCGAG<br>ATGCAGCCTGTAGAAGAAACGCAGGACGGCGAATAAAAGCTTGGCTGTTTT
**AP_GF33**	AGCGAATTCGAGCTCAGCAGGAGGAATTCAATGCGTGCTTTACCGATCTGTTTAGTAG<br>CACTCATGCTAAGCGGCTGTTCCATGTTAAGCAGATCCCCTGTCGAACCCGTTCAAA<br>GCACTGCACCCCAGCCGAAAGCGGAGCCTGCAAAACCGAAAGCGCCGCGCGCCA<br>CGCCGGTCCGAATTTATACCAATGCAGAAGAATTAGTCGGCAAACCGTTCCGCGATC<br>TCGGTGAAGTCAGTGGCGACTCTTGCCAGGCCTCTAATCAGGACTCTCCGCCGAGC<br>ATTCCAACCGCACGTAAGCGGATGCAAATCAACGCCTCTAAAATGAAAGCCAATGCT<br>GTATTACTGCATAGCTGCGAAGTCACCAGCGGTACGCCAGGCTGCTATCGTCAGGCT<br>GTATGTATCGGTTCTGCGCTTAACATTACGGCGAAATGAAAGCTTGGCTGTTTT

**AP_GJ1**	CGCCAATCGATATAAAGCAGGAGGAATTCAATGCAGTATTGGGGAAAAATCATTGGCG TGGCCGTGGCCTTACTGATGGGCGGCGGCTTTTGGGGCGTAGTGTTAGGCCTGTTAA TTGGCCATATGTTTGATAAAGCCCGTAGCCGTAAAATGGCGTGGTTCGCCAACCAGC GTGAGCGTCAGGCGCTGTTTTTTGCCACCACTTTTGAAGTGATGGGGCATTTAACCAA ATCCAAAGGTCGCGTCACGGAGGCTGATATTCATATCGCCAGCCAGTTGATGGACCG AATGAATCTTCATGGCGCTTCCCGTACTGCGGCGCAAAATGCGTTCCGGGTGGGAAA ATCAGACAATTACCCGCTGCGCGAAAAGATGCGCCAGTTTCGCAGTGTCTGCTTTGG TCGTTTTGACTTAATTCGTATGTTTCTGGAGATCCAGATTCAGGCGGCGTTTGCTGATG GTTCACTGCACCCGAATGAACGGGCGGTGCTGTATGTCATTGCAGAAGAATTAGGGA TCTCCCGCGCTCAGTTTGACCAGTTTTTGCGCATGATGCAGGGCGGTGCACAGTTTG GCGGCGGTTATCAGCAGCAAACTGGCGGTGGTAACTGGCAGCAAGCGCAGCGTGG CCCAACGCTGGAAGATGCCTGTAATGTGCTGGGCGTGAAGCCGACGGATGATGCGA CCACCATCAAACGTGCCTACCGTAAGCTGATGAGTGAACACCATCCCGATAAGCTGG TGGCGAAAGGTTTGCCGCCTGAGATGATGGAGATGGCGAAGCAGAAAGCGCAGGAA ATTCAGCAGGCATATGAGCTGATAAAGCAGCAGAAAGGGTTTAAATGACTAAGTAATAT GGTG
**AP_GJMF**	CGCCAATCGATATAAAGCAGGAGGAATTCAATGGATGTCATTAAAAAGAAACATTGGTG GCAAAGCGACGCGCTGAAATGGTCAGTGCTAGGTCTGCTCGGCCTGCTGGTGGGTT ACCTTGTTGTTTTAATGTACGCACAAGGGGAATACCTGTTCGCCATTACCACGCTGATA TTGAGTTCAGCGGGGCTGTATATGTTTGATAAAGCCCGTAGCCGTAAAATGGCGTGGT TCGCCAACCAGCGTGAGCGTCAGGCGCTGTTTTTTGCCACCACTTTTGAAGTGATGG GGCATTTAACCAAATCCAAAGGTCGCGTCACGGAGGCTGATATTCATATCGCCAGCC AGTTGATGGACCGAATGAATCTTCATGGCGCTTCCCGTACTGCGGCGCAAAATGCGT TCCGGGTGGGAAAATCAGACAATTACCCGCTGCGCGAAAAGATGCGCCAGTTTCGC AGTGTCTGCTTTGGTCGTTTTGACTTAATTCGTATGTTTCTGGAGATCCAGATTCAGGCG GCGTTTGCTGATGGTTCACTGCACCCGAATGAACGGGCGGTGCTGTATGTCATTGCA GAAGAATTAGGGATCTCCCGCGCTCAGTTTGACCAGTTTTTGCGCATGATGCAGGGC GGTGCACAGTTTGGCGGCGGTTATCAGCAGCAAACTGGCGGTGGTAACTGGCAGCA AGCGCAGCGTGGCCCAACGCTGGAAGATGCCTGTAATGTGCTGGGCGTGAAGCCG ACGGATGATGCGACCACCATCAAACGTGCCTACCGTAAGCTGATGAGTGAACACCAT CCCGATAAGCTGGTGGCGAAAGGTTTGCCGCCTGAGATGATGGAGATGGCGAAGCA GAAAGCGCAGGAAATTCAGCAGGCATATGAGCTGATAAAGCAGCAGAAAGGGTTTAA ATGACTAAGTAATATGGTG

Supplementary References:

1. Baba T, Ara T, Hasegawa M, Takai Y, Okumura Y, Baba M, et al. Construction of Escherichia coli K-12 in-frame, single-gene knockout mutants: the Keio collection. Mol Syst Biol. 2006;2:2006.0008. Epub 20060221. doi: 10.1038/msb4100050. PubMed PMID: 16738554; PubMed Central PMCID: PMCPMC1681482.
2. Karimova G, Pidoux J, Ullmann A, Ladant D. A bacterial two-hybrid system based on a reconstituted signal transduction pathway. Proc Natl Acad Sci U S A. 1998;95(10):5752-6. doi: 10.1073/pnas.95.10.5752. PubMed PMID: 9576956; PubMed Central PMCID: PMCPMC20451.
3. Brill JA, Quinlan-Walshe C, Gottesman S. Fine-structure mapping and identification of two regulators of capsule synthesis in Escherichia coli K-12. J Bacteriol. 1988;170(6):2599-611. doi: 10.1128/jb.170.6.2599-2611.1988. PubMed PMID: 2836365; PubMed Central PMCID: PMCPMC211177.
4. Majdalani N, Hernandez D, Gottesman S. Regulation and mode of action of the second small RNA activator of RpoS translation, RprA. Mol Microbiol. 2002;46(3):813-26. doi: 10.1046/j.1365-2958.2002.03203.x. PubMed PMID: 12410838.
5. Majdalani N, Heck M, Stout V, Gottesman S. Role of RcsF in signaling to the Rcs phosphorelay pathway in Escherichia coli. J Bacteriol. 2005;187(19):6770-8. doi: 10.1128/jb.187.19.6770-6778.2005. PubMed PMID: 16166540; PubMed Central PMCID: PMCPMC1251585.
6. Cabrera JE, Jin DJ. Growth phase and growth rate regulation of the rapA gene, encoding the RNA polymerase-associated protein RapA in Escherichia coli. J Bacteriol. 2001;183(20):6126-34. doi: 10.1128/jb.183.20.6126-6134.2001. PubMed PMID: 11567013; PubMed Central PMCID: PMCPMC99692.
7. Sharan SK, Thomason LC, Kuznetsov SG, Court DL. Recombineering: a homologous recombination-based method of genetic engineering. Nat Protoc. 2009;4(2):206-23. doi: 10.1038/nprot.2008.227. PubMed PMID: 19180090; PubMed Central PMCID: PMCPMC2790811.
8. Svenningsen SL, Costantino N, Court DL, Adhya S. On the role of Cro in lambda prophage induction. Proc Natl Acad Sci U S A. 2005;102(12):4465-9. Epub 20050223. doi: 10.1073/pnas.0409839102. PubMed PMID: 15728734; PubMed Central PMCID: PMCPMC555511.
9. Yahashiri A, Babor JT, Anwar AL, Bezy RP, Piette EW, Arends SJR, et al. DrpB (YedR) Is a Nonessential Cell Division Protein in Escherichia coli. J Bacteriol. 2020;202(23). Epub 20201104. doi: 10.1128/jb.00284-20. PubMed PMID: 32900831; PubMed Central PMCID: PMCPMC7648144.
10. Wall EA, Majdalani N, Gottesman S. IgaA negatively regulates the Rcs Phosphorelay via contact with the RcsD Phosphotransfer Protein. PLoS Genet. 2020;16(7):e1008610. Epub 20200727. doi: 10.1371/journal.pgen.1008610. PubMed PMID: 32716926; PubMed Central PMCID: PMCPMC7418988.
11. Thompson KM, Rhodius VA, Gottesman S. SigmaE regulates and is regulated by a small RNA in Escherichia coli. J Bacteriol. 2007;189(11):4243-56. Epub 20070406. doi: 10.1128/jb.00020-07. PubMed PMID: 17416652; PubMed Central PMCID: PMCPMC1913397.
12. Battesti A, Tsegaye YM, Packer DG, Majdalani N, Gottesman S. H-NS regulation of IraD and IraM antiadaptors for control of RpoS degradation. J Bacteriol. 2012;194(10):2470-8. Epub 20120309. doi: 10.1128/jb.00132-12. PubMed PMID: 22408168; PubMed Central PMCID: PMCPMC3347191.

13. Gibson DG, Young L, Chuang RY, Venter JC, Hutchison CA, 3rd, Smith HO. Enzymatic assembly of DNA molecules up to several hundred kilobases. Nat Methods. 2009;6(5):343-5. Epub 20090412. doi: 10.1038/nmeth.1318. PubMed PMID: 19363495.
14. Guzman LM, Belin D, Carson MJ, Beckwith J. Tight regulation, modulation, and high-level expression by vectors containing the arabinose PBAD promoter. J Bacteriol. 1995;177(14):4121-30. doi: 10.1128/jb.177.14.4121-4130.1995. PubMed PMID: 7608087; PubMed Central PMCID: PMCPMC177145.
15. Cherepanov PP, Wackernagel W. Gene disruption in Escherichia coli: TcR and KmR cassettes with the option of Flp-catalyzed excision of the antibiotic-resistance determinant. Gene. 1995;158(1):9-14. doi: 10.1016/0378-1119(95)00193-a. PubMed PMID: 7789817.
16. Karimova G, Ullmann A, Ladant D. Protein-protein interaction between Bacillus stearothermophilus tyrosyl-tRNA synthetase subdomains revealed by a bacterial two-hybrid system. J Mol Microbiol Biotechnol. 2001;3(1):73-82. PubMed PMID: 11200232.
17. Karimova G, Dautin N, Ladant D. Interaction network among Escherichia coli membrane proteins involved in cell division as revealed by bacterial two-hybrid analysis. J Bacteriol. 2005;187(7):2233-43. doi: 10.1128/jb.187.7.2233-2243.2005. PubMed PMID: 15774864; PubMed Central PMCID: PMCPMC1065216.
18. Clarke DJ, Holland IB, Jacq A. Point mutations in the transmembrane domain of DjlA, a membrane-linked DnaJ-like protein, abolish its function in promoting colanic acid production via the Rcs signal transduction pathway. Mol Microbiol. 1997;25(5):933-44. doi: 10.1111/j.1365-2958.1997.mmi528.x. PubMed PMID: 9364918.
19. Guillier M, Gottesman S. Remodelling of the Escherichia coli outer membrane by two small regulatory RNAs. Mol Microbiol. 2006;59(1):231-47. doi: 10.1111/j.1365-2958.2005.04929.x. PubMed PMID: 16359331.
20. Mandin P, Gottesman S. Integrating anaerobic/aerobic sensing and the general stress response through the ArcZ small RNA. Embo j. 2010;29(18):3094-107. Epub 20100803. doi: 10.1038/emboj.2010.179. PubMed PMID: 20683441; PubMed Central PMCID: PMCPMC2944060.
